# Serious infections in JIA patients upon MTX, TFN inhibitors and combinations

**DOI:** 10.1186/1546-0096-12-S1-O11

**Published:** 2014-09-17

**Authors:** Gerd Horneff, Ingrid Becker

**Affiliations:** 1 Asklepios Clinics, Sankt Augustin, Germany; 2 Institute of Medical Statistics, Cologne, Germany

## Introduction

Serious infections are a major concern in JIA patients treated with immunosuppressants and biologics. Effect of TNF inhibitors on the risk for serious infections and further factors are studied here.

## Methods

Pts exposed to Etanercept (ETA), Adalimumab (ADA) and Methotrexate (MTX) but no biologics and serious infections were identified in the BIKER registry. Descriptive statistics, infection rates, Cox-regression, Hazard ratios (HR) were calculated. Potential risk factors were analysed.

## Results

A total of 3350 pts. with 5929 exposure years were identified in the German BIKER registry data base. First biologic was ETA in 1720 and ADA in 177 cases. 1353 patients were not exposed to biologics. 28 serious infections have been reported (4.7/1000 pt-years). MTX patients had 5 events, Etanercept patients 21 and patients with Adalimumab therapy 2 events.The serious infections were of bacterial origin in 16, viral in 10 and unknown in 2. Total infection incidence per 1000 person years was 4.72 (CI 3.26 – 6.84). The highest rate was found under ADA (9.73, CI 2.43-38.91) followed by ETA (8.08, CI 5.27 – 12.40) and the lowest rate of 1.6 (CI 0.67 – 3.84) with MTX. Univariate Cox regression revealed a number of significant risks for infection, beside therapy (p=0.004) also JADAS10 at start of therapy, mean JADAS level during therapy, corticosteroids as pre- or concomitant medication as well as MTX and DMARDS as premedication were relevant. In multivariate Cox regression only therapy and mean JADAS10 during observation time remained significant. Both ETA (HR=4.88) and ADA (HR=10.06) showed an increased risk compared to MTX, whereas ADA and ETA differed not significally. Risk for infection was significantly increased by an elevated mean JADAS10 level (HR=1.12). Gender, JIA category, age at start of disease, disease duration, ANA status were not significant. Kaplan Meier analysis confirmed the influence of disease activity as demonstrated by the JADAS10. Table 1.

**Table 1:** 

Variable	univariate	HR (CI)	multivarite	HR (CI)
ADA / MTX	p=0.022	7.28(1.32-40.04)	p00.019	10.06(1.67-60.67)

ETA / MTX	p=0.001	5.96 (2.03-17.48)	p=0.013	4.88(1.39-17.08)

More than 1 DMARD failure	p=0.006	3.06 (1.39-6.75)		

Pretreatment with steroids	p=0.007	3.02(1.34-6.77)		

Steroids at baseline	p=0.026	0.418 (0.19-0.90)		

MTX concomitant	p=0.001	5.54(2.08-14.71)		

JADAS baseline	p=0.004	1.09 (1.03-1.16)		

Mean JADAS-level	p<0.001	1.12(1.05-1.19)	p<0.001	1.12(1.05-1.19)

## Conclusion

ETA and ADA treatment as well as higher disease activity contribute to serious infections.

## Disclosure of interest

None declared.

